# Effects of 8-Prenylnaringenin and Whole-Body Vibration Therapy on a Rat Model of Osteopenia

**DOI:** 10.1155/2016/6893137

**Published:** 2016-01-19

**Authors:** Daniel B. Hoffmann, Markus H. Griesel, Bastian Brockhusen, Mohammad Tezval, Marina Komrakova, Bjoern Menger, Marco Wassmann, Klaus Michael Stuermer, Stephan Sehmisch

**Affiliations:** ^1^Department of Trauma and Reconstructive Surgery, University of Goettingen, 37075 Goettingen, Germany; ^2^Medical Institute of General Hygiene and Environmental Health, University of Goettingen, 37075 Goettingen, Germany

## Abstract

*Background*. 8-Prenylnaringenin (8-PN) is the phytoestrogen with the highest affinity for estrogen receptor-*α* (ER-*α*), which is required to maintain BMD. The osteoprotective properties of 8-PN have been demonstrated previously in tibiae. We used a rat osteopenia model to perform the first investigation of 8-PN with whole-body vertical vibration (WBVV).* Study Design*. Ovariectomy was performed on 52 of 64 Sprague-Dawley rats. Five weeks after ovariectomy, one group received daily injections (sc) of 8-PN (1.77 mg/kg) for 10 weeks; a second group was treated with both 8-PN and WBVV (twice a day, 15 min, 35 Hz, amplitude 0.47 mm). Other groups received either only WBVV or no treatment.* Methods*. The rats were sacrificed 15 weeks after ovariectomy. Lumbar vertebrae and femora were removed for biomechanical and morphological assessment.* Results*. 8-PN at a cancer-safe dose did not cause fundamental improvements in osteoporotic bones. Treatment with 8-PN caused a slight increase in uterine wet weight. Combined therapy using WBVV and 8-PN showed no significant improvements in bone structure and biomechanical properties.* Conclusion*. We cannot confirm the osteoprotective effects of 8-PN at a cancer-safe dose in primary affected osteoporotic bones. Higher concentrations of 8-PN are not advisable for safety reasons. Adjunctive therapy with WBVV demonstrates no convincing effects on bones.

## 1. Introduction

In developed countries, postmenopausal osteoporosis is currently a serious problem that will only escalate in the future. Multiple prognoses and aging populations indicate that there will be a significant increase (more than 100%) in typical osteoporotic fractures, such as proximal femur fractures, over the next several decades [1]. The main cause of postmenopausal osteoporosis is estrogen deficiency, which increases bone resorption and accelerates bone loss [2]. Unfortunately, hormone replacement therapy (HRT), which prevents hip and spinal fractures, is no longer recommended following the 2002 Women's Health Initiative (WHI) study that revealed its life-threatening side effects, including the increased risk of cancer, stroke, and arteriosclerosis [3]. Therefore, safe and effective alternative therapies for osteoporosis are greatly needed.

Interest in phytoestrogens has recently increased. Phytoestrogens are hormonally active plant-derived compounds with estrogen-like effects on estrogen-dependent tissues [4]. More specifically, phytoestrogens interact directly with the *α* and *β* estrogen receptors (ERs) [4]. Both of these receptors are expressed in bone cells, including osteoclasts, osteoblasts, osteocytes, and chondrocytes [5]. ER-*α* is especially important for bone development and maintaining bone mineral density [6]. It has crucial effects on both trabecular and cortical bone [6]. ER-*α* acts on cells by stimulating target gene transcription through two activation functions (AF1 and AF2) [7]. Unlike ER-*α*-AF2, the ER-*α*-AF1 pathway is tissue-specific and essential for trabecular bone growth [7]. Additionally, ER-*β* only minimally influences cortical bone in mice, as reported previously [8]. Unfortunately, most of the known phytoestrogens primarily interact with ER-*β*.

Examples of mild osteoprotective phytoestrogens are soy, genistein, daidzein, equol, and 8-prenylnaringenin (8-PN) [4, 9, 10]. Genistein, daidzein, and equol demonstrate higher affinities for ER-*β* [11], whereas 8-PN has a higher affinity for ER-*α* [12]. 8-PN is a component of female hop cones, as well as of a crude Thai drug [13], and is therefore a component of beer. Recently, the beneficial effects of 8-PN as an herbal alternative for menopausal vasomotor complaints have been described [14]. Of all of the phytoestrogens, 8-PN has the highest affinity for the ER-*α* receptor [12] and is therefore an attractive molecule for osteoporosis research. Previous evidence has demonstrated the osteoprotective effects of 8-PN [4, 15, 16]. Unfortunately, in all of these studies, only the tibiae were investigated. There are no data on the osteoprotective effects of 8-PN in vertebrae or femora, which have a main role in osteoporosis. Furthermore, in these former studies, the dosage of 8-PN varied considerably. In some, high dosages were used to demonstrate osteoprotective effects [4, 16]. This dosage is not advisable because the risk for endometrial cancer is increased via an ER-*α* receptor-driven mechanism [17, 18]. From the oncological point of view, the dosage should be as low as possible even for the phytoestrogen 8-PN. An osteoprotective treatment with 8-PN is only reasonable if there is no increased risk of cancer. Thus, we wanted to investigate the effects of 8-PN on femora and vertebrae at a safe dose [16].

In addition to pharmaceutical therapy, mechanical stimulation is an alternative treatment for osteoporosis. Whole-body vibration is a safe and successful treatment [19]. According to the mechanostat model described by Frost, mechanical stimulation induces bone growth as a result of local elastic bone deformation [20]. Mechanical stress stimulates osteocytes, osteoblasts, and other cells of the bone lining to produce bone matrix via multiple pathways [21]. The use of vibration has been shown to increase both cortical and trabecular bone in animals [22].

The aim of this study was to evaluate the combined effects of 8-PN, an ER-*α* agonist, and whole-body vertical vibration (WBVV) as an osteoporosis treatment for the first time. We used the ovariectomized rat, which is a standard animal model for osteoporosis research [23]. Osteoporosis predominantly affects trabecular bones, such as the distal radius, femoral neck, and vertebral body. Because vertebral and femoral fractures are an important indicator of the progression of osteoporosis, lumbar vertebrae and femora were analyzed.

## 2. Materials and Methods

### 2.1. Animals and Substances

All of the procedures were approved by the local Institutional Animal Care and Use Committee (permission number 33.9-42502-04-12/0854, district authorities of Oldenburg, Germany). Consistent with recommendations in previous studies, experiments were performed on 64 three-month-old female Sprague-Dawley rats weighing 230–290 g (Fa. Winkelmann, Borchen, Germany) [23]. The rats were maintained according to German animal protection laws and fed a soy-free diet (ssniff Special Diet, Soest, Germany). A total of 52 rats were ovariectomized (*n* = 52) at the age of three months. The other 12 rats were not subjected to surgery (non-OVX, *n* = 12). After surgical treatment, the rats were divided into different groups.

The rats not operated on were placed in Group 1 (non-OVX). Ovariectomized rats receiving no treatment comprised Group 2 (OVX). Group 3 (OVX + VIB) contained ovariectomized rats treated with low-magnitude, high-frequency WBVV. Groups 4 (OVX-8-PN) and 5 (OVX-8-PN + VIB) received injections of 8-PN (Orgentis Chemicals GmbH, Gatersleben, Germany) five weeks after ovariectomy. Rats in Group 5 (OVX-8-PN + VIB) were treated with both 8-PN and WBVV.

For WBVV treatment, the rats were placed on a vibration platform twice daily for 15 min each, 5 days per week for 10 weeks beginning five weeks after ovariectomy. The vibration motor was constructed by Schultheis (Vibra Drehstrom-Vibrationsmotor Typ HVL/HVE, Offenbach, Germany), and it vibrated at a frequency of 35 Hz with a mean amplitude of 0.47 mm. Transmitted acceleration rate measured at the back of the rat was 0.2 g.

Rats treated with 8-PN received daily subcutaneous injections of 8-PN at a concentration of 1.77 mg/kg for ten weeks beginning five weeks after ovariectomy. 8-PN (purity > 98% by HPLC) was diluted in 30% hydroxypropyl-*β*-cyclodextrin (AppliChem GmbH, Darmstadt, Germany).

All animals were sacrificed under CO_2_-anesthesia at 15 weeks after ovariectomy. The lumbar vertebrae were removed for ashing for mineral content analysis (second lumbar vertebrae), biomechanical testing (third lumbar vertebrae), microcomputed tomography (fourth lumbar vertebrae), and gene expression analysis (sixth lumbar vertebrae). The vertebrae were stored in tubes at −20°C until the analyses were performed. For gene expression analysis, the samples were stored at −80°C.

### 2.2. Compression Test

**Figure 1:**
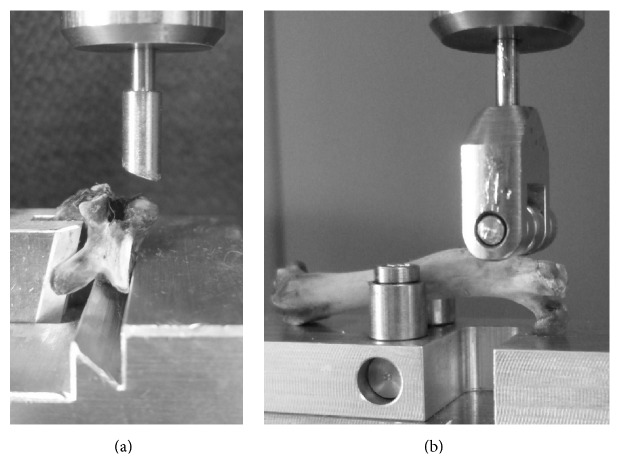
The thawed vertebrae (a) and femora (b) were fixed to the aluminum base, and the stamp was lowered with a primary force of 1 N to fix the bones at 50 mm/min. The range of the testing machine is 2–500 N.

Biomechanical tests were performed according to the protocol standardized by Sehmisch et al. [24]. A mechanical testing machine (Zwick, type 145 660 Z020/TND, Ulm, Germany) was used to measure the resistance of the lumbar vertebrae to force. The thawed vertebrae were fixed to the aluminum base (Figure 1(a)), and the stamp was lowered at a speed of 50 mm/min with a primary force of 1 N to fix the upper body plate. Measurements were obtained with a relative accuracy of 0.2–0.4% over the range of 2–500 N. The measurements were automatically stopped when the linear increase of the curve declined more than 10 N. Strength admission was recorded using testXpert software (Zwick, Ulm, Germany). The actual strength was measured in increments of 0.1 mm. We tested the femora in a similar way as described by Tezval et al. [25] (Figure 1(b)).

We quantified the maximum load (*F*_max_), yield load (yL), and stiffness (*S*) as described by Sehmisch et al. [10, 26]. The maximum load (*F*_max_) is the most force that the ground plate can withstand. The yield load (yL) is the inflection point from elastic deformation to plastic deformation. The stiffness measures the elasticity of the bone.

### 2.3. Microcomputed Tomography

An eXplore Locus SP microcomputed tomography scanner (GE Healthcare, Chalfont St Giles, UK) was used to analyze bone mineral density and other structural bone properties. Each scan included six vertebrae simultaneously. To compare the different scans, a test block was integrated into every scan. The test block consisted of five different materials with known mineral densities. To generate 3D models, GEHC Micro View v. 2.1.2 (GE Healthcare, Chalfont St Giles, UK) was used. We measured the following properties consistent with ASBMR nomenclature: trabecular thickness (Tb.Th), trabecular number (Tb.N), cortical thickness (Ct.Th), number of trabecular nodes (N.Nd), and bone volume fraction (BV/TV) [27, 28]. The vertebral body volume was calculated using the formula for a cylinder. For this calculation, the 2 cranial and 2 caudal perpendicular diameters and the dorsal and ventral heights were measured on the 3D images.

### 2.4. Microradiographic Analysis of Femora

We used microradiographic analysis to obtain more information about structural properties. For these tests, 150 *μ*m thick sagittal sections of femoral heads were used. The sections were cut out between the epiphyseal and intertrochanteric line. A Leica microscope (Leica-Systems MZ 7.5, Wetzlar, Germany) was used to measure the parameters. The pictures were digitalized by Qwin software (Leica, Wetzlar, Germany). We measured trabecular nodes (N.Nd), trabecular connectivity [N.Nd/mm^2^], trabecular bone area, trabecular thickness (Tb.Wi), trabecular density, and cortical density.

### 2.5. Ashing

The mass of mineralized bone from vertebrae was measured by ashing. The second lumbar vertebrae were heated in a muffle oven at 750°C for 30 min, and the bones were weighed to the nearest 0.00001 g before and after ashing. The mineral content (ash weight) is expressed relative to the wet weight of each vertebra (%).

The calcium content was assessed using an atomic absorption spectrometer (4100, PerkinElmer, Waltham, USA) according to CEN. The orthophosphate content was measured using a colorimetric method (ZeissDM4 spectrophotometer, Oberkochen, Germany) according to CEN.

### 2.6. Serum Analysis

Alkaline phosphatase (ALP) activity was measured in blood samples using an electrochemiluminescence immunoassay (Roche Diagnostics, Mannheim, Germany). The immunoassay was performed according to the manufacturer's instructions (Roche Diagnostics, Mannheim, Germany).

### 2.7. Gene Expression Analysis

For gene expression analyses, the sixth lumbar vertebrae were homogenized using a micro-dismembrator S (Sartorius, Göttingen, Germany). The RNeasy Mini Kit (Qiagen, Hilden, Germany) was used to extract the RNA, and the RNA was reverse-transcribed using Superscript RNase H-reverse transcriptase (Promega, Mannheim, Germany). The expression levels of alkaline phosphatase (ALP), receptor activator of nuclear factor *κ*B ligand (RANKL), osteocalcin, tartrate-resistant acid phosphatase (TRAP), and osteoprotegerin (OPG) were measured using quantitative real-time polymerase chain reaction (qRT-PCR) based on SYBR green detection (QuantiTect SYBR Green PCR Kit, Qiagen) in an iCycler (CFX96, Bio-Rad Laboratories, Munich, Germany). Primers from Qiagen (QuantiTect Primer Assays, Qiagen) were used, and quantitative real-time PCR was performed according to the manufacturer's instructions. Gene expression was calculated using the 2^ΔΔCT^ method [29], and the results shown are normalized to the gene expression in untreated female rats (non-OVX). The reference gene was *β*2-microglobulin.

### 2.8. Statistical Analysis

Data are shown as the means and standard deviation (SD). Significant differences were analyzed by one-way ANOVA with a Tukey-Kramer post hoc test (Graph Pad Prism, San Diego, USA). *p* values < 0.05 were considered significant.

## 3. Results

**Figure 2:**
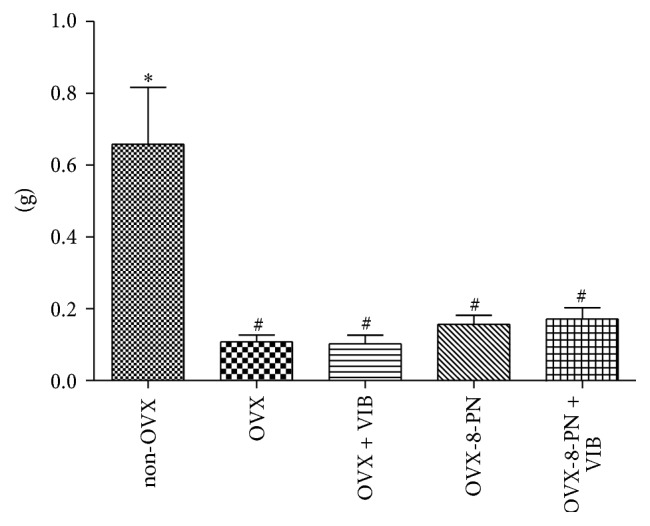
The uterine wet weight of ovariectomized rats treated with 8-PN increased nonsignificantly compared with ovariectomized rats that received no treatment. ^*∗*^ *p* < 0.05 versus OVX, ^#^ *p* < 0.05 versus non-OVX.

At the beginning of the study, the rats had approximately the same weights (260 ± 12.2 g). After the ovariectomy, typical changes in metabolism induced increases in body weight. The non-OVX rats also increased in body weight by the end of the evaluation period (15 weeks), which is consistent with normal growth. However, the non-OVX rats gained significantly less weight than the ovariectomized rats (Table 1). Only ovariectomized rats treated with 8-PN and WBVV (OVX-8-PN + VIB) demonstrated no significant increase compared with non-OVX rats. As expected, the uterine wet weight was highest in the non-OVX rats. Ovariectomized rats treated with 8-PN tended to have higher uterine wet weights than those of the other ovariectomized rats (Figure 2).

### 3.1. Biomechanical Assessment of Vertebrae

**Figure 3:**
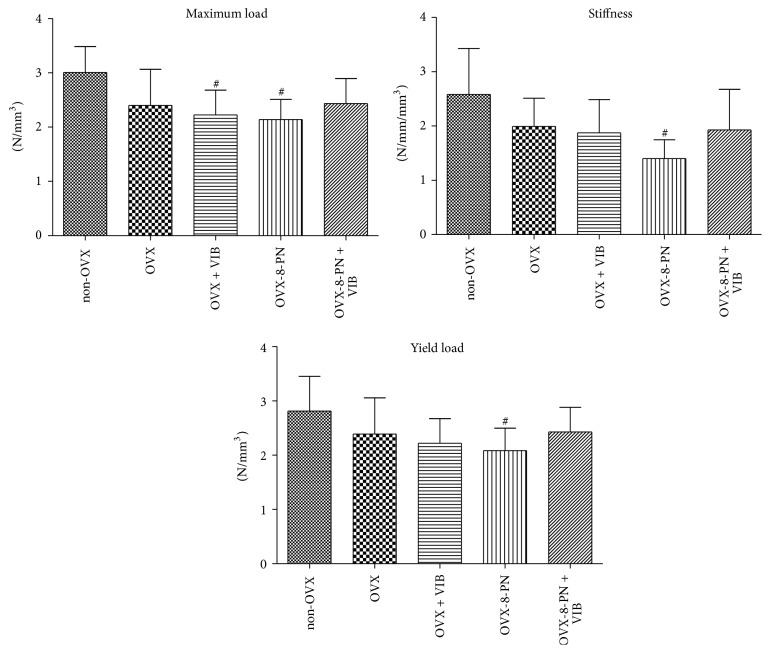
Biomechanical assessment of vertebrae (measurements normalized to the bone volume). Single therapy with 8-PN or WBVV induced in vertebrae a significant decrease in biomechanical properties compared with non-OVX rats. Compared with untreated ovariectomized rats, treatment with 8-PN tended to worsen biomechanical properties. Adjunctive therapy using 8-PN with WBVV produced no significant improvements. Non-OVX rats had the highest values for all of the biomechanical properties. Vertebrae measurements were normalized to the bone volume. ^#^ *p* < 0.05 versus non-OVX.

To exclude the effects of different vertebral body sizes and volumes, all *F*_max_, yL, and stiffness measurements were normalized to the bone volume determined in the micro-CT analysis [24]. In our study, treatment with 8-PN did not improve the biomechanical properties of vertebrae (Figure 3). In contrast, single therapy using 8-PN significantly worsened the biomechanical properties compared with those of non-OVX rats and tended to worsen these properties compared with untreated ovariectomized rats. Adjunctive treatment of WBVV and 8-PN caused no significant changes in the biomechanical properties. Single therapy with WBVV did not significantly affect the *F*_max_, yL, or stiffness compared with those of ovariectomized rats that received no treatment (Figure 3). Non-OVX rats demonstrated the best biomechanical results.

### 3.2. Biomechanical Assessment of Femora

**Figure 4:**
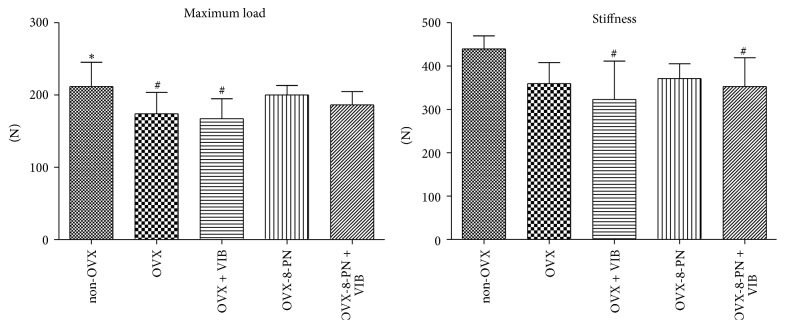
Biomechanical assessment of femora (absolute values): treatment with 8-PN as single therapy caused no significant decrease in the biomechanical properties compared with those of non-OVX rats. Compared with untreated ovariectomized rats, treatment with 8-PN showed only a nonsignificant improvement. There were no significant effects attributable to WBVV as a single or adjunctive therapy with 8-PN. ^*∗*^ *p* < 0.05 versus OVX, ^#^ *p* < 0.05 versus non-OVX.

For femora, absolute values were measured. In contrast to the results for the vertebrae, treatment with 8-PN as a single therapy caused no significant decrease in the biomechanical properties in the femora compared with those of non-OVX rats and a slight but nonsignificant increase compared with those of untreated ovariectomized rats (Figure 4). Dual therapy with WBVV and 8-PN caused no significant improvements in ovariectomized rats. There were no significant effects attributable to WBVV as a single or adjunctive therapy. Altogether, the results in the femora are consistent with the results shown in the vertebrae.

### 3.3. Microcomputed Tomography of Vertebrae

**Figure 5:**
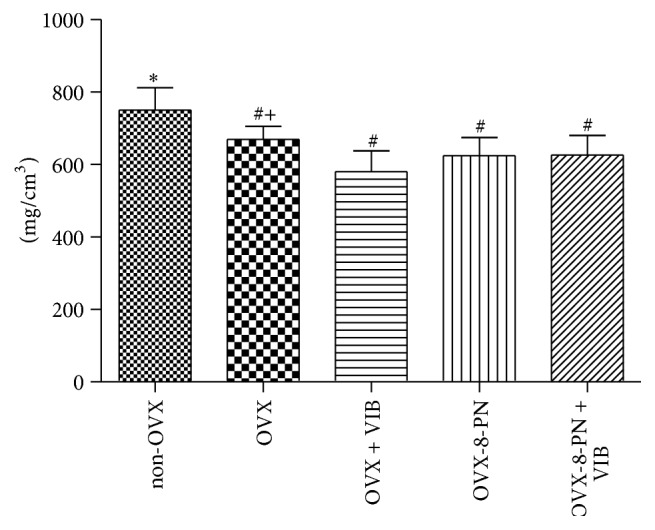
The BMD significantly decreased after treatment with WBVV compared with that in untreated ovariectomized rats. Neither treatment with 8-PN alone nor dual therapy with WBVV and 8-PN showed improving effects on the BMD of ovariectomized rats. Non-OVX rats had significantly higher BMD than that of all ovariectomized rats irrespective of any therapy. ^*∗*^ *p* < 0.05 versus OVX, ^#^ *p* < 0.05 versus non-OVX, and ^+^ *p* < 0.05 versus adjunctive VIB.

The bone mineral density (BMD) significantly decreased after treatment with WBVV compared with that in untreated ovariectomized rats (Figure 5). Neither treatment with 8-PN alone nor dual therapy with WBVV and 8-PN showed improving effects on the BMD of ovariectomized rats. Non-OVX rats had significantly higher BMD than that of all ovariectomized rats irrespective of any therapy. In the BV/TV of trabecular bone, treatment with 8-PN alone and as adjunctive therapy showed a slight increase but with no statistical effect. For the trabecular thickness (Tb.th), trabecular number (Tb.N), and cortical thickness (Ct.Th), no improving effects of 8-PN or WBVV were observed compared with the values in untreated ovariectomized rats.

### 3.4. Microradiographic Analysis of Femora

Neither vibration therapy nor treatment with 8-PN or adjunctive therapy had a significant effect on the structural bone properties in the femoral neck. Non-OVX rats demonstrated the best results.

### 3.5. Ashing of Vertebrae

The non-OVX rats had significantly higher mineral content than that of the ovariectomized rats. Compared with untreated rats, rats treated with WBVV (OVX + VIB, OVX-8-PN + VIB) had lower mineral contents (Table 1).

The Ca^2+^/PO_4_^3−^ ratios did not differ from ovariectomized rats.

### 3.6. Serum Analysis

Ovariectomized rats treated with WBVV alone had significantly higher concentrations of alkaline phosphatase (ALP) (OVX + VIB 149.6 ± 33.8 U/I) than that of ovariectomized rats that received no treatment (OVX 113.4 ± 18.3 U/I) (Table 1). Treatment with 8-PN alone (OVX-8-PN 137.4 ± 28.1 U/I) and dual therapy with WBVV (OVX-PN + WBVV 135.9 ± 8.4) also caused significantly increased ALP levels compared with that in non-OVX rats but with no statistical effects compared with untreated ovariectomized rats. Non-OVX rats had the lowest levels of ALP.

### 3.7. Gene Expression Analysis

The mRNA-expression of the bone-resorptive enzyme ALP significantly increased in the rats treated with WBVV and 8-PN as dual therapy compared with untreated ovariectomized rats (Table 1). Single treatment with 8-PN and WBVV resulted in a nonsignificant increase in ALP-mRNA. The non-OVX rats had the lowest expression of RANKL-mRNA. A nonsignificant increase in OPG expression was observed in ovariectomized rats following WBVV treatment (Table 1).

## 4. Discussion

Several recent studies have investigated vibration or phytoestrogen treatment as potential new therapies for osteoporosis [4, 9, 19, 30, 31]. Almost all phytoestrogens tested in previous studies predominately acted via the estrogen receptor ER-*β* [4, 10, 11, 32]. Unfortunately, this receptor only minimally affects bones. In contrast to ER-*β*, the estrogen receptor ER-*α* exerts crucial effects on trabecular and cortical bone [6]. Of all of the phytoestrogens, 8-PN has the highest affinity for ER-*α* [4, 12]. This property makes 8-PN unique and interesting. However, to date, there has only been limited research into the effects of 8-PN on osteoporosis and on bones in general [4, 15, 16]. Osteoprotective effects were only shown in tibia and not in femora or spine, which are predominately affected by osteoporosis. From the authors' point of view, conclusive data supporting the benefits of 8-PN as an osteoprotective drug in the case of osteoporosis are still lacking.

In the present study, we could not demonstrate fundamental improvements in osteoporotic vertebrae and femora after treatment with 8-PN. Neither the biomechanical nor the morphological properties improved significantly in our study. Instead, we could demonstrate that 8-PN nonsignificantly worsened the biomechanical properties and BMD in the vertebrae. In contrast to the bone data, a slight increase in uterine weight confirmed the systemic estrogen-like effects. Our results for the biomechanical and structural bone parameters in femora and vertebrae differ from those of previous studies. In 2008, Sehmisch et al. reported that 8-PN significantly improved the biomechanical properties of bone in tibiae. However, only minimal improvements in bone structure were observed [4]. In 1998, Miyamoto et al. showed an increase in BMD after the administration of 8-PN [15]. Similar results were shown by Hümpel et al. [16]. However, none of these studies tested femora or spine parameters. Additionally, the rates and methods of 8-PN administration differed considerably. The dosages in these previous studies differed from 1.77 mg/kg per day to 68 mg/kg per day [4, 15, 16]. This fact is important from a safety point of view. Even the 1.77 mg/kg dosage demonstrated a weak stimulation of endometrial luminal epithelial cells due to an ER-*α*-driven mechanism [16]. In the present study, we administered 8-PN subcutaneously at a dose of 1.77 mg/kg to determine whether a cancer-safe dose has beneficial effects on an osteoporotic spine and femora neck. We were unable to confirm this effect. From the authors' point of view, the administration of a higher 8-PN concentration to improve osteoprotective effects is not advisable due to safety reasons.

Mechanical stimulation by vibration therapy has shown beneficial effects on the structure and biomechanical properties of bones, including vertebrae, in several animal studies of osteoporosis [19, 22, 30, 33, 34]. A systemic meta-analysis in 2010 showed significant but small effects in postmenopausal women [33].

In the present study, we could not demonstrate significant improvements in the biomechanical properties and bone structure after WBVV. The effects of WBVV as a single or adjunctive therapy were more pronounced in previous studies [19, 22, 30, 31]. However, all of these studies were performed on different bones (tibia, femur, and lumbar vertebrae) and at varying frequencies. We used a frequency of 35 Hz and amplitude of 0.47 mm in our study based on our own previous and external studies [34–36]. Compared with ovariectomized rats that received no treatment, rats that received WBVV treatment showed higher bone turnover, as demonstrated by increased RANKL, ALP, and osteocalcin expression. However, overall, we could not confirm that beneficial effects were exerted on bones in our setting. In our opinion, the optimal setting including frequency, amplitude, and acceleration for WBVV has not yet been determined, and data in the literature for the rat osteopenia model are contradictory [31, 34, 35]. In the present study, only bone parameters were investigated. It is reasonable that muscle status can be improved by WBVV. Increased muscle strength is beneficial for preventing falls and maintaining bone mass. Further studies are needed to study the integral effects of vibration therapy in the case of osteoporosis.

Adjunctive therapy using WBVV and the ER-*α* agonist 8-PN has not previously been investigated. The present study is the first to investigate a dual treatment using WBVV and 8-PN. No significant improvements in bone structure were observed. There were no effects on biomechanical properties. According to our results, adjunctive therapy with 8-PN and WBVV at 35 Hz has no effects on bones, which are predominately affected by osteoporosis.

## 5. Conclusion

In conclusion, we cannot confirm the osteoprotective effects of 8-PN at a cancer-safe dose in primary affected osteoporotic bones. In our opinion, higher concentrations of 8-PN are not advisable due to safety reasons. Adjunctive therapy with WBVV at 35 Hz has no significant effects on bones. Further studies are needed to investigate the integral effects and best setting of WBVV in the case of osteoporosis.

## Figures and Tables

**Table 1:** Body weight, *μ*CT, protein expression, and serum analysis data after treatment with WBVV, 8-PN, or combined therapy.

	non-OVX		OVX		OVX + VIB		OVX-8-PN		OVX-8-PN + VIB	
Sample size	11		11		11		11		12	
	Mean	STD	Mean	STD	Mean	STD	Mean	STD	Mean	STD
*Bodyweight*										
Pre-OVX [g]	258.0	8.9	264.3	13.8	258.3	11.8	264.1	10.8	257.5	14.8
After 15 weeks [g]	346.6^a^	19.1	400.7^c^	47.4	392.5^c^	24.0	388.3^c^	21.3	367.0	22.1
*µCT vertebrae*										
Trabecular number (Tb.N) [*n*]	111.5	26.98	117.5	25.56	105.4	27.27	117.6	25.45	113.8	27.91
Number of trabecular nodes N.Nd [*n*]	133.8	36.76	140.8	34.79	122.8	36.33	143.6	36.05	137.1	37.56
Mean trabecular junctions at one node (Tb.N/Nd) [*n*]	2.33	0.14	2.33	0.16	2.25	0.14	2.37	0.14	2.36	0.17
Trabecular thickness (Tb.Th) [mm]	0.05669	0.02143	0.04956	0.02006	0.04975	0.0138	0.04884	0.01932	0.04827	0.02725
BV/TV whole body of vertebrae [%]	43.82	3.22	39.84	1.94	42.66	4.46	41.68	5.82	43.32	2.92
BV/TV trabecular bone [%]	52.55	6.75	48.53	7.04	47.30	8.53	50.13	9.24	58.03	15.85
Cortical thickness (CTTh) [mm]	0.2367	0.0212	0.1977	0.0393	0.2116	0.0412	0.2042	0.0356	0.2021	0.0408
*Microradiographic analysis femora*										
Trabecular nodes (N.Nd) [*n*]	109.1	21.7	87.5	19.2	78.4^c^	23.1	87.0	8.7	91.6	9.9
Trabecular connectivity [N.Nd/mm^2^]	21.85^a^	3.93	16.97^c^	3.29	14.09^c^	4.13	16.32^c^	1.46	17.21^c^	2.87
Trabecular bone area [mm^2^]	4.99	0.45	5.16	0.70	5.62	0.69	5.33	0.28	5.39	0.64
Trabecular thickness (Tb.Wi) [mm]	0.00698	0.00171	0.00597	0.00079	0.00564^c^	0.00056	0.00553^c^	0.00071	0.00572^c^	0.00071
Trabecular density [%]	75.50^a^	11.77	58.50^c^	10.29	50.80^c^	10.75	53.80^c^	7.15	56.48^c^	9.99
Cortical density [%]	97.44	1.41	96.62	1.49	97.03	0.96	96.52	0.88	96.14	1.22
*Ashing*										
Mineral content (%)	43.00^a^	7.59	37.82^c^	1.66	35.97^c^	1.54	37.46	1.68	36.00^c^	2.10
Ca^2+^/PO_4_^3−^	1.541	0.065	1.500	0.220	1.562	0.156	1.609	0.065	1.569	0.031
*Gene expression*										
ALP	1.056	0.325	0.825	0.190	1.376	0.435	1.300	0.584	1.665^a^	0.238
Osteocalcin	1.114	0.574	1.631	0.549	2.066	1.085	1.142	0.471	1.306	0.537
RANKL	1.199	0.556	1.956	0.310	2.938^c^	0.874	2.054	0.633	1.661	0.816
OPG	1.218	0.968	1.296	0.732	1.595	1.066	1.472	1.756	2.187	1.801
TRAP	1.069	0.494	1.152	0.248	1.136	0.569	0.867	0.471	0.841	0.384
*Serum analysis*										
ALP [U/I] serum	91.4	15.37	113.4^b^	18.34	149.6^ac^	33.8	137.4^c^	28.07	135.9^c^	8.39

^a^ *p* < 0.05 versus OVX.

^b^ *p* < 0.05 versus adjunctive VIB.

^c^ *p* < 0.05 versus non-OVX.

## Abbreviations

8-PN: 8-Prenylnaringenin
ALP: Alkaline phosphatase
BV/TV: Bone volume fraction
BMD: Bone mineral density
Ct.Th: Cortical thickness
ER: Estrogen receptor
*F*_max_: Maximum load
HRT: Hormone replacement therapy
N.Nd: Number of trabecular nodes
N.Nd/mm^2^: Trabecular connectivity
OPG: Osteoprotegerin
RANKL: Receptor activator of nuclear factor *κ*B ligand
*S*: Stiffness
Tb.N: Trabecular number
Tb.Th: Trabecular thickness
yL: Yield load.
